# Task-Specific Lingual Dystonia During Japanese Religious Services

**DOI:** 10.7759/cureus.50115

**Published:** 2023-12-07

**Authors:** Kazuya Yoshida

**Affiliations:** 1 Department of Oral and Maxillofacial Surgery, National Hospital Organization, Kyoto Medical Center, Kyoto, JPN

**Keywords:** genioglossus muscle, oromandibular dystonia rating scale, sensory trick splint, speech-induced dystonia, muscle afferent block therapy, tongue, onabotulinumtoxina, botulinum toxin therapy, task-specificity, lingual dystonia

## Abstract

Introduction: Lingual dystonia is a subtype of oromandibular dystonia characterized by involuntary contractions of the tongue muscles, often provoked by speaking or eating.

Methods: This study reports six Japanese cases (four female and two male, mean age at onset of 49.5 years) with task-specific lingual dystonia during praying. In the early phase, all patients experienced lingual protrusion exclusively during Japanese religious services. When the patients start speaking, the tongues protrude forward, making it difficult to pronounce words. The patients were treated with multimodal treatment, including muscle afferent block (MAB) therapy comprising local anesthetic injection, botulinum toxin (onabotulinumtoxinA) injection, and a sensory trick splint.

Results: MAB therapy was conducted in five patients (mean time: 5.8), and botulinum toxin injection was administered in four patients (mean time: 8). The injected muscles were the genioglossal muscles and, in one case, the lateral pterygoid muscle. Sensory trick splints were inserted in three patients. After the multimodal therapy, the patients were able to pronounce words smoothly and clearly. Oromandibular Dystonia Rating Scale scores improved significantly (P<0.005) from baseline (187 points) to endpoint (47 points) with a mean follow-up of 4.7 years.

Conclusion: Although this entity is rare, medical and dental professionals should be aware of this peculiar symptom. Multimodal therapy is required to ensure effective treatment of praying-induced lingual dystonia.

## Introduction

Oromandibular dystonia (OMD) is a focal dystonia characterized by contractions of the masticatory or lingual muscles [1,2]. Lingual dystonia is a subtype of OMD that shows a task-specific etiology, with symptoms often developing during speech or mastication [3,4]. In the early phase, dystonia is generally associated with a specific task [4,5], with symptoms later extending to other tasks, and eventually occurring even at rest. Symptoms of task-specific dystonia often develop during the performance of a specific skilled motor task [4]. Speech-induced lingual dystonia is reported to be associated with professions in which conversations in stressful situations are unavoidable [4]. Several cases of praying-induced OMD or lingual dystonia have previously been reported in the literature, with symptoms provoked exclusively when patients recited Quranic verses in Arabic [6-8]. The present study reports six cases of lingual dystonia with symptoms occurring in Japanese religious services.

## Materials and methods

Herein, the author presents six patients (four female and two male, with a mean age at onset of 49.5 years) with praying-induced lingual dystonia associated with Japanese religious services. The demographic and clinical characteristics of these patients are summarized in Table 1. Only one Shinto priest was religious by profession, and all other patients were Buddhists with non-religious occupations (Table 1). The patients showed considerable individual differences in the time they attended services and the period until symptom onset (Table 1). All patients exhibited task specificity, stereotypy, and sensory tricks while chewing gum, eating food, or using a handkerchief (Table 1). The severity, disability, psychosocial functioning, impact on the quality of life, and therapeutic changes were evaluated using the Oromandibular Dystonia Rating Scale (OMDRS) at baseline and treatment endpoints [9]. The severity scale, disability scale, pain scale, total examiner-rated scale, and total scores of OMDRS (summation of total examiner-rated scale and total patient-rated questionnaire scores) are shown in Table 1. In the early phase, all patients experienced lingual protrusion exclusively during Japanese religious services. However, the average duration between disease onset and the first visit was approximately four years (Table 1). Symptoms tended to gradually appear during overall speech. When the patient starts speaking, the tongue protrudes forward, making it difficult to pronounce words. Patient 3 received etizolam and clotiazepam prior to disease onset (Table 1), but the remaining patients had no history of neuroleptic or dopamine-blocking agent exposure.

**Table 1:** Demographics and clinical characteristics of the patients SD: standard deviation, M: male, F: female, OMDRS: Oromandibular Dystonia Rating Scale

Patient	Age at onset (years)	Duration (months)	Sex	Occupation	Religious service/day (minutes)	Period of religious service (months)	Sensory tricks	OMDRS at baseline				
								Severity scale	Disability scale	Pain scale	Examiner-rated scale	Total OMDRS
1	34	24	M	Shinto priest	180	120	Chewing gum	6	7	0	13	128
2	53	7	M	Salesperson	50	516	Chewing gum	7	13	0	20	161
3	64	8	F	Salesperson	30	360	Chewing gum	12	17	8	37	244
4	44	156	F	Architect	30	360	Chewing gum	7	4	0	11	147
5	54	48	F	Care worker	30	480	Foods, handkerchief	11	10	26	47	229
6	48	48	F	Housewife	300	2	Chewing gum	10	15	7	32	213
Mean (SD)	49.5 (10.2)	48.5 (55.7)	-	-	103.3 (112.7)	306.3 (203.6)	-	8.8 (2.5)	11 (4.9)	6.8 (10.1)	26.3 (14.7)	187 (47.9)

This study was performed in accordance with the Declaration of Helsinki after obtaining approval from the Institutional Review Board and Ethics Committee of the Kyoto Medical Center (approval number: 15-031). The patients received a detailed explanation of the planned treatment and publication of the results and provided written informed consent.

## Results

**Figure 1:**
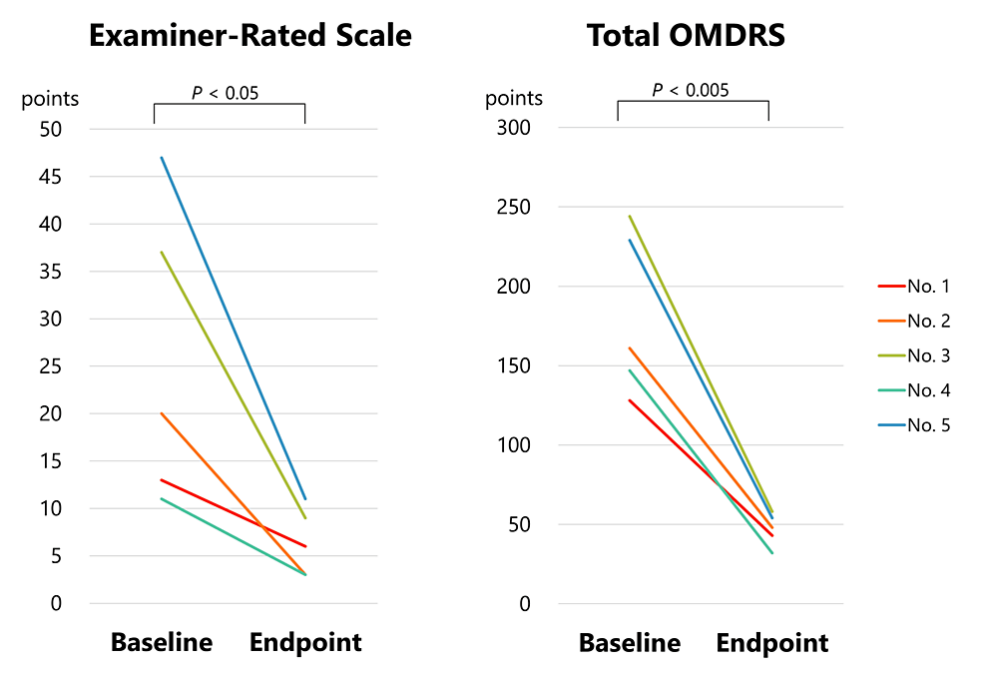
Changes in OMDRS scores from baseline to endpoint The total examiner-rated scale (0-86 points) is the sum of the severity scale (0-16 points), disability scale (0-30 points), and pain scale (0-40 points), and the total OMDRS (0-314 points) is the sum of the total examiner-rated scale and the total patient-rated questionnaire score (0-228 points)

Treatment results are summarized in Table 2. Oral medications, such as baclofen, trihexyphenidyl, and clonazepam, were prescribed without satisfactory effects (Table 2). However, muscle afferent block (MAB) therapy with a local anesthetic (0.5% lidocaine) injection was administered to five patients 35 times (Table 2), as previously described [1,2]. Botulinum toxin (onabotulinumtoxinA) was administered 48 times in four patients (Table 2), as reported previously [2,10]. All patients received injections in the genioglossal muscles, with an additional injection in the lateral pterygoid muscle in one case. Sensory trick splints were inserted in three patients (Table 2) [11]. After the multimodal therapy, the patients were able to pronounce words smoothly and clearly. Most of the scores reduced significantly from baseline to endpoint (severity, 8.8 to 2.2, P<0.005, paired t-test; disability, 11 to 2.2, P<0.02; total examiner-rated scale scores, 26.3 to 6.4, P<0.05; and total OMDRS scores, 187 to 47, P<0.005) (Tables 1-2, Figure 1). The endpoint was the time when the patient was satisfied with the therapeutic effect and botulinum toxin therapy was completed. Pain scores reduced from 6.8 to 2.4; however, this difference was not significant (P=0.25). The mean follow-up was 4.7 years.

**Table 2:** Results of treatment of the patients MAB: muscle afferent block, OMDRS: Oromandibular Dystonia Rating Scale

Patient	Oral medication	MAB	Botox	Injected muscles	Sensory trick splint	Follow-up (month)	OMDRS at endpoint				
							Severity scale	Disability scale	Pain scale	Examiner-rated scale	Total OMDRS
1	Baclofen	5	0	Genioglossus	+	23	3	3	0	6	43
2	Trihexyphenidyl, clonazepam	11	17	Genioglossus	+	105	1	2	0	3	48
3	Baclofen, clonazepam	17	5	Genioglossus, lateral pterygoid	+	35	3	4	2	9	58
4	Clonazepam	1	11	Genioglossus	-	87	2	1	0	3	32
5	-	1	15	Genioglossus	-	86	2	2	7	11	54
6	Trihexyphenidyl, haloperidol	0	0	-	-	3	-	-	-	-	-
Mean (SD)	-	5.8 (20.5)	8 (7.4)	-	-	56.5 (41.5)	2.2 (0.8)	2.2 (1.1)	2.4 (2.9)	6.4 (3.6)	47 (10.2)

## Discussion

This is the first report of praying-induced lingual dystonia during Japanese religious services, although a similar association with the recitation of Islamic prayers has been reported previously. This study supports the proposal that multimodal therapy is necessary and effective in the treatment of pray-induced lingual dystonia.

OMD is believed to be a rare disease; however, this may be due to under- or misdiagnosis. However, a recent study reported the prevalence of OMD to be 9.8 per 100,000 individuals [12]. This suggests that OMD is not rare but is rarely properly diagnosed or treated. As the symptoms of OMD can considerably vary by subtype, it is difficult to comprehensively measure disease severity and changes after treatment [9]. Recently, the OMDRS was developed and validated [9]. This scale can be useful in the comprehensive evaluation of severity, disability, psychosocial functioning, impact on quality of life, and therapeutic changes [9].

The Prajnaparamita Sutra has always been recited as scripture in several Buddhist sects. Although there are various schools of thought among the different sects, recitation of the Prajnaparamita Sutra every morning and evening is obligatory in all. However, as this text is written in Chinese, Japanese people are generally unable to understand its exact meaning. Similarly, the Quran is written in Arabic, meaning that Muslims whose mother tongue is not Arabic may experience similar situations. Daily repetition of foreign language scriptures is considered a significant source of stress and may induce lingual dystonia. All eight cases of lingual dystonia associated with praying reported so far have been Muslims who were not native speakers of Arabic [6-8].

Ginatempo et al. [13] reported that the integration between sensory input and motor output is disrupted at the cortical level with topographic specificity in OMD. Isolated focal task-specific dystonia impairs not only motor dexterity but also somatosensory perception in well-trained and skilled motor tasks [4,5]. There are two dominant hypotheses regarding the underlying mechanisms of focal task-specific dystonia: impaired inhibition and abnormal plasticity regulation [4]. A previous study on 95 patients with speech-induced lingual dystonia found that 82.1% of the patients had engaged in occupations that required them to talk to other people in stressful situations over prolonged periods of time for many years [4]. The most common occupation was sales representative (17.9%), followed by telephone operator (13.7%), customer service representative (10.5%), healthcare worker (9.5%), waiter or waitress (5.3%), receptionist (5.3%), and cashier (5.3%) [4].

Illic et al. [6] previously reported the case of a 47-year-old man of Turkish descent with a complaint of “slurred speech” when reciting Islamic prayers in Arabic. Dystonic contraction of the left-sided perioral muscles can only be induced by praying in Arabic. They recommended that the patient use the sensory trick maneuver to alleviate the symptoms. Kutty et al. [7] similarly reported six patients (three females and three males with ages ranging from 39 to 71 years) with lingual dystonia associated with reciting the Quran. In five patients, this lingual dystonia was exclusively present when reciting Quranic verses in Arabic. One patient also had mild OMD while speaking in the native language (Malay), which worsened upon reciting the Quran. All patients had lingual dystonia of varying severity associated with jaw tightness (n=6), blepharospasm (n=2), and additional spasmodic dysphonia (n=2) upon progressive recitation. Most patients showed minimal improvement with oral medications. Three patients received botulinum toxin injections with minimal improvement. Salari et al. [8] reported the case of a 36-year-old man with difficulty pronouncing the sounds “s” and “z” only while praying when he would try to pronounce Arabic phenomes correctly. After one year, he subsequently developed intermittent lingual dystonia, which affected his overall speech. Speech therapy and pharmacotherapy were attempted but were ineffective. The patient showed mild improvement in a trial involving tetrabenazine and trihexyphenidyl. After a year, he had sustained difficulty pronouncing all sounds, but the problems related to sounds were more severe.

OMD treatment must be multimodal and individualized. Therapeutic options may include botulinum toxin therapy, oral medication, MAB therapy, sensory trick splints, and surgery [2]. Botulinum toxin therapy is considered the first choice of treatment for lingual dystonia [2,3,14,15]. An experienced clinician would carefully select target muscles and injection sites and determine the dose and allocation for each botulinum toxin injection, corresponding to patient symptoms and the results of palpation and electromyographic measurements [2]. Personalized adjustment of target muscles, sites, and doses will result in significantly improved outcomes compared with standard methods without individualized planning [2,3]. In a recent study, the target lingual muscles were examined in detail and injected using individualized methods, which achieved a higher success rate and a lower rate of adverse events [3]. Of the eight cases reported to date, only three patients were treated with botulinum toxin therapy with minimal improvement [6]. In this report, four patients were treated with botulinum toxin injections with very good responses. Botulinum toxin therapy of the tongue muscle has previously been shown to be effective for lingual dystonia, with few side effects [3].

Unfortunately, previously reported patients with this condition were not managed adequately and satisfactorily. Furthermore, the objective and follow-up data were not described. The average time delay between the onset of the disease and the visit to our department was approximately four years, during which time the patient's symptoms worsened. If the disease could be diagnosed and treated as soon as possible, then therapeutic effects could be improved further. Although this entity is extremely rare, medical and dental professionals should be aware of this peculiar symptom, which appears to present exclusively during religious rituals.

This retrospective study has some limitations. First, this study was uncontrolled in an open-label fashion. Second, the small sample size may have been inadequate for statistical analyses. Hence, although this condition is very rare, further studies with more samples are necessary to obtain significant evidence.

## Conclusions

This study reports six Japanese cases of task-specific lingual dystonia during praying. All patients experienced lingual protrusion exclusively during Japanese religious services. MAB therapy and botulinum toxin therapy were conducted on the genioglossal muscles. OMDRS scores improved significantly from baseline to endpoint. Multimodal therapy is required to ensure effective treatment of praying-induced lingual dystonia.
